# Effect of nursing intervention via a chatting tool on the rehabilitation of patients after Total hip Arthroplasty

**DOI:** 10.1186/s13018-019-1483-4

**Published:** 2019-12-09

**Authors:** Jing Luo, Xiaohua Dong, Jing Hu

**Affiliations:** 1 Department of Nursing Administration, Honghui Hospital, Xi’an Jiaotong University College of Medicine, 555# You-yi East Road, Xi’an, 710054 Shaanxi People’s Republic of China; 2 Department of Cardiovascular, Honghui Hospital, Xi’an Jiaotong University College of Medicine, Xi’an, People’s Republic of China

**Keywords:** Nursing intervention, Elderly patients, WeChat, Total hip arthroplasty, Rehabilitation

## Abstract

**Background:**

Nursing intervention following discharge is a long-term rehabilitation process that is essential for improving hip joint function and quality of life in affected patients. This study aimed to assess the effect of nursing intervention via WeChat on the rehabilitation of patients after total hip arthroplasty (THA).

**Methods:**

We conducted a retrospective analysis of 232 patients who underwent THA at our hospital from January 2013 to October 2015. Of the 232 patients, 114 received nursing intervention via telephone (Group A), and 118 received nursing intervention via WeChat (Group B). Furthermore, the Harris hip score and Short-Form 36 (SF-36) health survey score were used to evaluate hip joint function and quality of life in patients in the two groups at discharge and 1, 3 and 6 months following discharge. Moreover, the functional independence measure was applied to assess the recovery of joint function in the patients.

**Results:**

No significant difference was observed in the Harris hip score and the SF-36 health survey score between the two groups at discharge and 1 month following discharge (*p* > 0.05). However, the Harris hip score and SF-36 health survey score were lower in group A than in group B at 3 and 6 months following discharge (*p* < 0.05). Furthermore, no obvious difference was observed in terms of functional independence between the two groups at discharge (*p* > 0.05). However, more individuals were completely independent in group B than in group A at 1, 3 and 6 months following discharge (*p* < 0.05).

**Conclusions:**

Nursing intervention via WeChat can improve the effect of rehabilitation after THA and promote the recovery of joint function in patients.

## Background

Hip joint diseases have become increasingly common with the increase in average life expectancy and significantly influence the quality of life in elderly individuals [1, 2]. To date, total hip arthroplasty (THA) has been an effective treatment for hip joint diseases and can successfully relieve pain, restore hip joint function, and improve the performance of daily activities [3, 4]. However, patients who undergo THA must actively perform rehabilitation exercises under the supervision of nurses. Because of the actual limitations of age, educational level, cognitive ability, and other issues, traditional rehabilitation guidance via telephone can be challenging for elderly individuals. Furthermore, in clinical practice, rehabilitation exercises, used to consolidate the curative effect of surgery, are still mainly performed during hospitalization in most areas in China. Patients and their family members have limited awareness regarding rehabilitation training after discharge, and the importance of such training is not well emphasized; this results in poor compliance after discharge in patients who undergo THA.

Nursing intervention following discharge is a long-term rehabilitation process that is essential for improving hip joint function and quality of life in affected patients [5, 6]. In recent decades, WeChat has been adopted as an extended nursing method following discharge and has been popularized and applied gradually in clinics [7, 8]. Disease-related knowledge can be sent to a specific population via WeChat using voice messages and video storage; this can strengthen health education and encourage patients to participate in the self-management of diseases. This approach is convenient and rapid and plays an important role in social communication. WeChat is a reliable method for patients to obtain timely health guidance and counseling, and it has achieved satisfactory results in clinical practice [7, 8]. However, limited reports have investigated the application of extended nursing after THA via WeChat. Accordingly, the present study aimed to assess the effect of nursing intervention via WeChat on the rehabilitation of patients after THA.

## Methods

### Patient population

We conducted a retrospective analysis including 232 patients who underwent THA at our hospital from January 2013 to October 2015. The enrolled patients had to be able to be contacted via telephone after discharge and personally use WeChat or had to have family members that could send the WeChat content to the patient in a timely manner. Other inclusion criteria were as follows: patients with joint dysfunction because of various causes requiring THA, those aged > 65 years with normal cognitive function and normal abilities of communication, those who volunteered to participate in the study and signed informed consent, and those with junior high school-level education or higher. The exclusion criteria were as follows: patients with mental disorders and serious chronic diseases in the heart, lung and brain; those who could not take care of themselves or those who had been involved in a similar experiment before. The study protocol was approved by the ethics review committee of Honghui Hospital, Xi’an Jiaotong University College of Medicine (2012–0024), and all patients provided written informed consent to participate in the study and the use and publication of data for research purposes.

### Grouping and interventions of the study

The enrolled patients were divided into two groups. In group A, the patients were provided with nursing intervention via telephone (which included THA-related knowledge, psychological support, dietary guidance, matters requiring attention after discharge, prevention of complications and rehabilitation guidance). In group B, the patients were provided with nursing intervention via WeChat. The interview content was sent via WeChat (the same content as that provided via telephone) to all patients.

While receiving routine rehabilitation guidance, the patients were provided with out-of-hospital rehabilitation guidance according to the contents of the procedures sent via telephone (group A) and WeChat (group B). Rehabilitation guidance mainly included the following contents: 1) 2–4 weeks after surgery: muscle strength training, range of motion training, wake-up activities, bed activities, sitting exercises, standing exercises, walking exercises and upstairs and downstairs exercises; 2) 5–12 weeks after surgery: muscle resistance training, range of motion training, weight-bearing and walking training and wearing shoes and socks. After discharge, the patients who underwent THA were followed-up once a week within the first month, once every 2 weeks from the second to third months and once a month from the fourth to sixth months (approximately 30 min for each follow-up).

Before participating in this study, all members of the study received unified training and were required to be qualified to perform the training through examination. The assessment included rehabilitation training and questionnaire filling guidance for patients who underwent THA at different stages following discharge.

### Assessment of hip joint function

Postoperative hip joint function was evaluated using the Harris hip scale, [9] which includes scores for pain, function, range of motion and deformity. Function included seven items, which were as follows: wearing shoes and socks, need for walking aids, sitting on a chair, entering the car, limping, walking distance and climbing stairs. Each item was assigned a different score, and the final score was summarized and categorized into the following four grades: 90–100 points, excellent; 80–89 points, good; 70–79 points, moderate; and < 70 points, poor.

### Assessment of quality of life

The Short-Form 36 (SF-36) questionnaire was used to assess the quality of life in patients [10]. SF-36 is a self-administered generic questionnaire measuring physical and emotional function and general health (GH) perception. The scale measures the two dimensions of health on eight subscales, which reflect the impact of both dysfunctions and GH perceptions. Dysfunctions were measured using the following subscales: physical function, physical role, bodily pain, social function, and emotional role. Health perception was measured with three subscales: GH, vitality, and mental health. The responses correlated to each subscale were transformed into a score on a scale from 0 (lowest score) to 100 (highest score), with a higher score indicating a better health status or absence of limitations.

### Assessment of functional Independence

The functional independence measure (FIM) scale was applied for comprehensively evaluating functional independence [11] and the therapeutic effect at each stage to determine the amount of nursing or time required by patients, to guide nursing work, and to evaluate the effect of the rehabilitation. The assessment criteria were as follows: 7 points, completely independent; 6 points, assisted independence; and 2 points, completely dependent. The FIM total score ranged from 18 (lowest score) to 126 points (highest score), and it was graded into three levels: totally independent (108–126 points), partially dependent (54–107 points) and totally dependent (18–54 points).

### Statistical analysis

The Statistical Package for the Social Sciences software version 21.0 was used for the statistical analysis of all data. Measurement data were expressed as X¯[[]] ± *S* (means ± standard deviations), which were examined using *t*-test and repeated measures analysis of variance. In addition, categorical data were analyzed using the χ^2^ test. A *P* value of < 0.05 was considered statistically significant.

## Results

In total, 114 patients in group A and 118 patients in group B were enrolled for subsequent analysis. In group A, 29.9% of patients were male, with a mean age of 73.9 years. However, in group B, 30.3% of patients were male, with a mean age of 72.5 years. No significant difference was observed in terms of gender, age, surgery duration, volume of blood loss and monthly income between the two groups, which indicates that the groups were comparable (Table 1).

**Table 1:** Comparison of basic characteristics between groups

Items	Group A (*n* = 114)	Group B (*n* = 118)
Gender (male)	35 (29.9%)	36 (30.3%)
Age (years)	73.9 ± 12.9 (65–87)	72.5 ± 13.1 (64–85)
Operation time (min)	90.5 ± 10.0 (81–125)	91.6 ± 14 (76–132)
Blood loss (ml)	320.1 ± 29 (250–500)	310.3 ± 32 (253–550)
Living alone	55 (48.3%)	61 (51.7%)
Dependent on help	35 (30.7%)	36 (30.5%)
Monthly income		
*<* 200 US	15 (13.2%)	16 (13.6%)
200–500 US	78 (68.4%)	79 (66.9%)
> 500 US	21 (18.4%)	23 (19.5%)

Data are presented as *n* (%) or mean ± standard deviation (range)

No significant difference was observed in the Harris hip score between the two groups at discharge and 1 month following discharge (*p* > 0.05). However, with an extended discharge time, the Harris hip score in the patients increased gradually, with the rate of increase in group B faster than that in group A. Furthermore, the Harris hip score in group B was significantly higher than that in group A at 3 and 6 months following discharge (*p* < 0.05; Table 2).

**Table 2:** Comparison of Harris hip scores between groups

Groups	N	At discharge	1 month following discharge	3 months following discharge	6 months following discharge
Group A	114	61.10 ± 9.42	69.1 ± 6.3	74.3 ± 9.6	82.54 ± 7.46
Group B	118	62.32 ± 8.25	70.0 ± 6.5	80.5 ± 8.9*	91.24 ± 9.08*

^*^p < 0.05, Comparison between groups A and B

No significant difference was observed in the SF-36 health survey scores between the two groups at discharge and 1 month following discharge (*p* > 0.05). However, at discharge, the SF-36 health survey score of the two groups increased gradually, and the rate of increase in group B was faster than that in group A. At 3 and 6 months following discharge, the score in group B was remarkably higher than that in group A (*p* < 0.05; Table 3).

**Table 3:** Comparison of Short Form-36 health survey scale scores between groups

Groups	n	At discharge	1 month following discharge	3 months following discharge	6 months following discharge
Group A	114	387.4 ± 83.2	465.1 ± 76.0	584.1 ± 85.6	670.6 ± 89.5
Group B	118	391.9 ± 92.0	468.0 ± 86.0	632.4 ± 98.0*	725.3 ± 86.5*

^*^p < 0.05, Comparison between groups A and B

No significant difference was observed in terms of functional independence between the two groups after discharge (*p* > 0.05). Significantly more people were completely independent in group B than in group A 1, 3 and 6 months following discharge (*p* < 0.05; Table 4).

**Table 4:** Comparison of functional independence between groups

Groups	N	At discharge			1 month following discharge			3 months following discharge			6 months following discharge		
		Complete dependence	Partial dependence	Complete independence	Complete dependence	Partial dependence	Complete independence	Complete dependence	Partial dependence	Complete independence	Complete dependence	Partial dependence	Complete independence
Group A	114	44	60	10	6	92	16	1	80	33	0	10	104
Group B	118	43	64	11	5	90	23*	0	60*	58*	0	1*	117*

^*^p < 0.05, Comparison between groups A and B

## Discussion

In addition to the success of the surgery, postoperative long-term rehabilitation nursing may be an essential determinant of the effect of rehabilitation after THA. In the course of home-based rehabilitation after THA, poor compliance among the patients may consequently result in unsatisfactory hip joint function recovery and poor quality of life. Inpatient nursing must be recognized as merely a part of holistic nursing, and nursing intervention after discharge is extremely important for joint function recovery [12].

With advantages such as convenience, rapidity and strong maneuverability, WeChat is used for extended nursing of discharged patients [7, 8]. With regard to the superiority of WeChat, it can assist patients in understanding the dynamics of drug use and rehabilitation exercise at home; monitor whether patients consume their medicine in a timely manner and perform rehabilitation training according to the discharge instructions; remind patients to maintain a proper diet; improve the patients’ self-care ability; effectively prevent various complications; and provide necessary emotional support to patients. Furthermore, based on the extension of the proposed nursing service, patients can actively adjust and arrange their mood, diet, lifestyle habits and functional exercises. If patients have questions and experience discomfort, they can communicate with professionals in a timely manner to seek solutions, which may be conducive to immediately resolving the condition and preventing unfavorable factors affecting rehabilitation. Therefore, using the extended nursing intervention model via WeChat can improve the compliance of patients after surgery, guide patients to develop function and regain health, effectively address possible challenges encountered by patients in home-based nursing, promote rehabilitation and improve quality of life [7, 8]. Our study validated the abovementioned results and showed that joint function recovery in group B was significantly greater than that in group A. In terms of potential causes, elderly individuals cannot grasp a large amount of medical information in a timely manner because of their old age and poor memory. At present, a telephone is considered to be the major approach to understanding the status of extended nursing in patients following discharge. With regard to education, a telephone provides elderly patients with oral education, which cannot provide timely and effective health education. In contrast, a WeChat is more flexible in terms of text, pictures, videos, and other intuitive methods that can immediately and accurately transmit health education through the mobile phones of the patients after hospital discharge, providing a convenient tool for understanding the information given to them.

In this study, the SF-36 health survey was used to evaluate the quality of life in patients with regard to psychological, social and emotional functions. Patients are at risk of adverse psychological and emotional effects that may decrease their compliance to a long-term rehabilitation process after THA. A WeChat can offer necessary emotional support to patients, improve patients’ moods to promote mental health, remind patients of their diet, supervise patients in their consumption of medicines in a timely manner and in performing rehabilitation training according to the procedures, improve patients’ self-care ability, and effectively prevent various complications. In the present study, no significant difference was observed in the SF-36 health survey scores between groups B and A at discharge and 1 month following discharge. At 3 and 6 months following discharge, the SF-36 health survey score in group B was higher than that in group A; this result indicated that extended nursing intervention via WeChat could significantly improve the quality of life in patients after THA.

A WeChat can enhance the ability of patients to live independently with its convenient, rapid, economical, educative and acceptable characteristics. The findings of this study revealed that the complete independence rate of patients in group B was significantly higher than that in group A at 3 months following discharge, and this finding supports the role of WeChat in decreasing dependence among elderly individuals. The improvement may be because patients with good joint recovery and an excellent joint score can share their experience and conduct peer education via WeChat. Counseling, support, comfort, help, encouragement and other measures from peers may alleviate the negative emotions of some patients, guide patients to have a positive attitude, and further build up confidence and courage to overcome pain. However, no significant difference was observed between the independence of patients in groups B and A at 1 month following discharge, which may be because of the long period required for patients in the observation group to fully recover their hip joint function after THA. Thus, the advantages of WeChat cannot be fully reflected in a very short time period. Notably, a WeChat can fulfill the requirements for patients in terms of education, improve the effect of education and promote their rehabilitation. However, because a WeChat is a recent, modern development, elderly patients cannot fully enjoy the convenience of the service because of cultural limitations. Therefore, the target population of this study comprised elderly individuals who can use WeChat independently, those who have a higher degree of education and those who rely on family members for health education via WeChat.

## Conclusions

Following discharge, nursing intervention via WeChat can improve hip joint function recovery, quality of life and functional independence in patients who undergo THA. Moreover, it can improve the effect of rehabilitation after THA.

## Data Availability

The datasets supporting the conclusions of this article are included within the article. The raw data can be requested from the corresponding author on reasonable request.

## Abbreviations

FIM: functional independence measure
SF-36: Short-Form 36
THA: total hip arthroplasty
